# A deep residual attention–Recurrent model for early and multi-stage Alzheimer's disease detection

**DOI:** 10.3389/fnagi.2026.1798861

**Published:** 2026-06-26

**Authors:** Uma Maheswara Rao Munipalli, Visalakshi Annepu

**Affiliations:** School of Computer Science and Engineering, VIT-AP University, Amaravathi, Andhra Pradesh, India

**Keywords:** Alzheimer's disease, Bi-GRU, CBAM, classification, mild cognitive impairment, neurodegenerative disorders, ResNet152V2

## Abstract

**Introduction:**

Alzheimer's disease (AD) is a progressive neurodegenerative disorder that requires accurate and early diagnosis. Deep learning methods have shown significant potential for automated MRI-based AD classification.

**Methods:**

A hybrid deep learning framework integrating ResNet152V2, Convolutional Block Attention Module (CBAM), and Bidirectional Gated Recurrent Unit (Bi-GRU) was developed for three-class classification of Normal Cognition (NC), Mild Cognitive Impairment (MCI), and Alzheimer's Disease (AD). The model was trained using 2,100 ADNI subjects and externally validated using 900 OASIS subjects.

**Results:**

The proposed framework achieved 94.5% classification accuracy with an AUC of 95.0% on the ADNI dataset and 92.8% accuracy on the OASIS dataset. Comparative analyses demonstrated improvements of 5.2–7.0% over baseline models. Ablation studies confirmed the contribution of CBAM and Bi-GRU to overall performance.

**Discussion:**

The integration of deep residual feature extraction, attention-based refinement, and sequential modeling effectively captures disease-related anatomical patterns. The results suggest that the proposed framework may support automated multi-stage Alzheimer's disease classification and provide a foundation for future computer-aided diagnostic systems.

## 1 Introduction

Alzheimer's disease (AD) is the major cause of 60–70% of dementia cases worldwide. It is the leading cause of the recent increase in attention to this medical diagnosis and has also developed a significant public health crisis (Chaudhary et al., 2024). The World Health Organization estimates that globally, the number of individuals suffering from AD and other dementias by 2050 is expected to be 152 million (Petersen et al., 2014). AD is characterized by a continuous and permanent progression of memory degradation, cognitive function, and loss of ability to perform daily activities. Alzheimer's disease develops in three stages: Normal Cognition (NC), Mild Cognitive Impairment (MCI), and Alzheimer's Dementia (AD). It has been identified that MCI serves as a crucial time frame in which treatment intervention could have an impact (Dubois et al., 2014; Frisoni et al., 2010). Clinical and neuropsychological assessments comprise the majority of patient analysis methods today, but neither approach adequately recognizes early-stage AD or distinguishes it from other forms of dementia with sufficient sensitivity. Therefore, structural MRI has emerged as a valuable neuroimaging biomarker because it can characterize unique patterns of brain atrophy, including cortical thinning and reduced size of the hippocampus (Litjens et al., 2017; Esteva et al., 2019). However, the manual interpretation of an MRI scan is inherently subjective and requires substantial time and effort, and this maintains the requirement for a consistent method of automating the diagnostic process.

Although some traditional machine learning paths, such as Support Vector Machines (SVM) and Random Forest (RF) classifiers, have been used to classify Alzheimer's Disease (AD), these methods depend mostly on manually created features. The barrier to handcrafted features is the capacity of these models to identify the complex and non-linear spatial characteristics of high-dimensional neuroimaging data. The deep learning models implement end-to-end learning of features directly from the raw imaging data. CNNs have been mostly used in the field of medical image processing with their flexibility and the capability to learn from the raw images.CNN architectures such as ResNet152V2 are effective in learning hierarchical spatial representations from neuroimaging data.

Most current MRI-based methodologies that use convolutional neural networks (CNNs) treat individual MRI slices as separate entities. This approach fails to capture the volumetric context and continuity between the slices owing to the spatial distribution of unique characteristics of neurodegenerative diseases associated with AD. An efficient approach to capturing these subsequent relationships between the slices of an MRI is the use of recurrent neural networks (RNNs), most importantly the Bidirectional Gated Recurrent Units (Bi-GRUs), which enable the encoder to gather information from both forward and backward motion. The hybrid CNN-RNN models that were developed previously, such as the CNN-long short-term memory (LSTM) model, needed to address this particular limitation, but were often unable to model both spatial and sequential data effectively, and they also lacked the strong attention mechanisms for clinically highlighting important anatomical regions (Slimi et al., 2025; Komal et al., 2025).

This study provides a thorough and powerful hybrid deep learning model which combines three distinct and key components: (1) a Bi-GRU neural network, which captures the inter-slice spatial–temporal dependence patterns which represent progressive neurodegeneration; (2) a Convolutional Block Attention Module (CBAM), which allows the selective emphasis of disease-relevant anatomical features; and (3) ResNet152V2 for extracting the deep hierarchical spatial features. The proposed architectural model combines deep residual learning, attention-based feature refinement, and bidirectional sequential modeling to improve the early identification of Alzheimer's disease and increase the overall classification performance by providing additional anatomical focus and volumetric context.

To address these challenges, this study proposes a hybrid framework that combines deep residual learning, attention-based feature refinement, and sequential modeling to effectively capture both spatial and inter-slice relationships in MRI data. Unlike conventional approaches that treat slices independently, the proposed method leverages a 2.5D representation to balance computational efficiency with anatomical context.

Unlike conventional approaches that treat slices independently, the proposed method leverages a 2.5D representation to balance computational efficiency with the anatomical context.

These findings can be summarized as follows:

- This study develops a new type of hybrid deep learning framework for classifying multiple stages of Alzheimer's disease, which uses structural MRI data and consists of three different parts: ResNet152V2, convolutional block attention module (CBAM), and bidirectional gated recurrent units (Bi-GRUs).
- This study introduces a new 2.5D spatial-sequential learning method, in which slices are extracted from the data using a deep residual network; these spatial features are then modeled across slices using Bi-GRU to effectively represent inter-slice anatomical relationships and to provide an efficient representation of the data.
- The research also introduces an attention-based feature refinement process for the model that uses CBAM to make sure that the model is focusing on clinically significant areas of the brain, thereby improving accuracy and providing more informative output.
- This study has conducted numerous experiments to validate its findings, including ablation studies, statistical tests with *p*-values (paired *t*-tests, McNemar's tests, and bootstrap confidence intervals), and investigations of demographic bias across groups.
- The study externally validates the proposed model using the OASIS database to test whether the proposed model would produce similar results in different patient cohorts.
- The research performs a balanced evaluation of model accuracy and computational complexity, emphasizing the trade-off between accuracy and efficiency in comparison to existing CNN-based, RNN-based, and transformer-based techniques.

The new model creates an efficient implementation of attention-based feature refinement and bidirectional sequential learning by introducing the former prior to sequentially modeling the images. Attention-based feature refinement makes it possible to obtain better quality representations for each image while also being efficient from a computational perspective.

This paper is organized as follows: Section 2 reviews related work, Section 3 describes the methodology, Section 4 details the proposed architecture, Section 5 presents the results, and Section 6 discusses the clinical implications.

## 2 Related work

Recent advancements in deep learning have resulted in hybrid systems that improve how we can utilize MRI images to diagnose Alzheimer's disease (AD) through automated means. A system developed an architecture combining convolutional neural networks (CNN) with transformers. This architecture utilized 2D dense neural networks to extract volumetric and spatial features from cross-sectional MRIs and 3D dense neural networks for volumetric and longitudinal features in serial imaging.

As a result, the CNN-transformer system has produced better volumetric feature extraction, spatial hierarchies, and interpretability compared with traditional CNN-based image processing methods, while also yielding excellent accuracy and performance metrics (Shourie et al., 2023; Sorour et al., 2024; Fathi et al., 2024). Another development of note is the creation of a system integrating a convolutional block attention module (CBAM) with capsule networks, known as CAPCBAM, to preserve spatiotemporal hierarchies while emphasizing the most important anatomical structures in both early and late coexisting phases of AD. CAPCBAM also demonstrated excellent performance in predicting the early phases of AD, achieving near-best precision and recall, and outperforming traditional CNN imaging systems regardless of age (Tanveer et al., 2022; Chen et al., 2025; Zheng et al., 2025).

However, these advancements are mindful of the mundane reality of data variability, deficient technology, and the presence of socio-economic inequities in healthcare that can hinder diagnostic accuracy and effectiveness. New approaches that are novel and transdisciplinary to overcome these barriers to care have included new biomarker discovery and artificial intelligence (AI)-assisted multimodal developments. Recent multimodal efforts that concurrently assess MRI and positron emission tomography (PET) modalities have begun to develop dual attentional (multi-head and cross-attention) mechanisms to better represent intermodal features (Rezaee and Zhu, 2025; Sharma and Meena, 2025; Chen et al., 2024). In these models, CNNs are tasked with comparing multi-scale features, while cross-modality fusion modules aggregate and build complements from the sub-nets that accompany each modality. Models that incorporate imaging, clinical, and demographic data incorporates DenseNets as an alternative to support the enhanced propagation of features and “Inception” models to augment multi-scale features for the detection of subtle brain irregularities (Altinkaya et al., 2020; Adjei et al., 2024; Al Olaimat et al., 2023).

In addition, explainable artificial intelligence (XAI) methods, such as Gradient-weighted Class Activation Mapping (Grad-CAM) and Shapley Additive Explanations (SHAP), have been adopted to enhance the transparency of our model and clarity for clinical interpretation. Recent advances in self-supervised learning and federated learning allow models to be pretrained on unlabelled data and collaborate across institutions, while preserving data privacy. All of these advances were brought together in our hybrid model, which uses ResNet152V2 as the main backbone, along with CBAM and Bi-GRU, to demonstrate a strong capacity for modeling spatial-temporal dependencies, along with increasing interpretability and extensibility in multimodality for future use in the early diagnosis of AD (Wu et al., 2022; Zhang et al., 2024; Cheng et al., 2024).

Accurate prediction of the development of Alzheimer's disease (AD) involves a multifactorial problem with biological variability, limitations of technology, and barriers to access to the medical field. Current research demonstrates that it is necessary for researchers to use combined integrative methods through the use of biomarker discovery, artificial intelligence, and the ability to use multiple types of data to increase accuracy in diagnosis. In the area of combining the data from MRI and PET scan studies, multimodal architectures demonstrate significant promise by integrating dual and cross-head attention in order to improve the alignment of intermodal feature representation and by including CNN modules to compare features at different scales, as well as using fusion modules to associate corresponding merging layers from both models to improve overall interpretation (Selvi et al., 2023; Lavanya et al., 2024; Zhang et al., 2022).

Recent hybrid models combining ResNet152V2, CBAM, and Bi-GRU have shown major advancements in both accuracy and interpretability. ResNet152V2 allows the spatial categorization of deeper and more complex image patterns, whereas CBAM reduces the distractibility of spatial-channel attention in CBAM to help focus attention on important brain features. Such hybrid architectures also include methods of temporal reasoning, such as Bi-GRU, which are more conducive to longitudinal imaging studies designed using large-cohort MRI study databases (Arafa et al., 2024; Hatami et al., 2025; Zhang et al., 2025). In addition to the above, hybrid architectures have been used in testing lightweight networks (e.g., ShuffleNet V1) that employ Efficient Channel Attention (ECA) methods to improve the robustness of models and generalizing to situations where there are limited data. A hybrid architecture that was found to improve metrics included Faster R-CNN to improve high-precision feature extraction (3D image levels) compared with Mobile Vision Transformer (MobileViT) architectures, which improved parameter efficiency and reduced the overall cost of computation while allowing the recognition of brain features through the use of MRIs (Mahavar et al., 2025; Alex et al., 2025; Petersen et al., 2024). Research utilizing residual learning and depthwise separable convolutions has also improved the ability to generalize and improve the efficiency of models, with accuracies exceeding 97%. Personalized Prediction of Alzheimer's Disease (PPAD) and PPAD-autoencoder models also extend the applicability of earlier models for predicting at-risk individuals for conversion from MCI to AD. Through density determination of volumetric MRI data, clustering methods have also identified several AD subtypes with unique subgroups and cognitive behaviors and may potentially provide additional insights into individualized treatment approaches (Basanta-Torres et al., 2025; Ren et al., 2025; Alasiry et al., 2025).

Table 1 presents a concise and well-structured synthesis of recent deep learning-based approaches for Alzheimer's disease diagnosis. It primarily focuses on architectural design choices, data modalities, and methodological limitations,, which are particularly valuable for deriving the proposed method.

**Table 1:** Comparative summary of recent deep learning-based methods for Alzheimer's disease diagnosis, highlighting architectural design choices, data modalities, principal contributions, and reported limitations.

References	Methodology	Modality	Key contributions	Limitations
(Chaudhary et al. 2024); (Chen et al. 2025); (Zhang et al. 2025)	Hybrid CNN-Transformer architectures	Structural MRI	Improved global feature modeling and enhanced classification accuracy compared to CNN-only models	High computational cost and reliance on large datasets
(Arafa et al. 2024); (Zhang et al. 2022)	Capsule networks with CBAM attention	Structural MRI	Preservation of spatial hierarchies and improved focus on salient anatomical regions	Model complexity and limited scalability
(Chen et al. 2023); (Zheng et al. 2025)	Ensemble CNN and CNN-RNN models	Structural MRI	Increased robustness via classifier aggregation	Reduced interpretability and higher training overhead
(Cheng et al. 2024); (Lavanya et al. 2024)	Attention-based multimodal fusion networks	MRI + PET	Effective intermodal feature alignment using attention mechanisms	Dependence on multimodal imaging availability
(Hatami et al. 2025)	DenseNet-based multimodal fusion frameworks	MRI + Clinical	Improved sensitivity to subtle neuroanatomical changes through multimodal integration	Challenges with data heterogeneity and imbalance
(Komal et al. 2025); (Ren et al. 2025)	Explainable CNN models (Grad-CAM, SHAP)	Structural MRI	Enhanced interpretability of model decisions	Inconsistent explanations across subjects
(Rezaee and Zhu 2025)	Lightweight hybrid CNN-Transformer models	Structural MRI	Reduced computational cost and improved generalization	Slightly reduced accuracy compared to deeper models
(Singh and Patel 2024); (Slimi et al. 2025)	AI-driven multi-omics integration frameworks	Imaging + Omics	Identification of novel biomarkers for personalized diagnosis	High-dimensional data complexity and limited availability

## 3 Methodology

This section presents the complete methodology of the proposed framework, including dataset preparation, preprocessing, model architecture, and training strategy. The overall pipeline is designed to extract meaningful spatial features from MRI slices, refine them using attention mechanisms, and model inter-slice dependencies for improved classification.

The methodology is organized into the following components:

(i) Dataset and preprocessing,
(ii) Experimental setup,
(iii) Proposed Hybrid Framework,
(iv) Spatial feature extraction,
(v) Attention-based Feature Refinement using CBAM,
(vi) Temporal Dependency Sequential modeling, and
(vii) Classification Layer.

### 3.1 Dataset and preprocessing

This research mainly used two publicly available MRI datasets, the Alzheimer's Disease Neuroimaging Initiative (ADNI) and Open Access Series of Imaging Studies (OASIS), to create and test the proposed framework.

**Figure 1:**
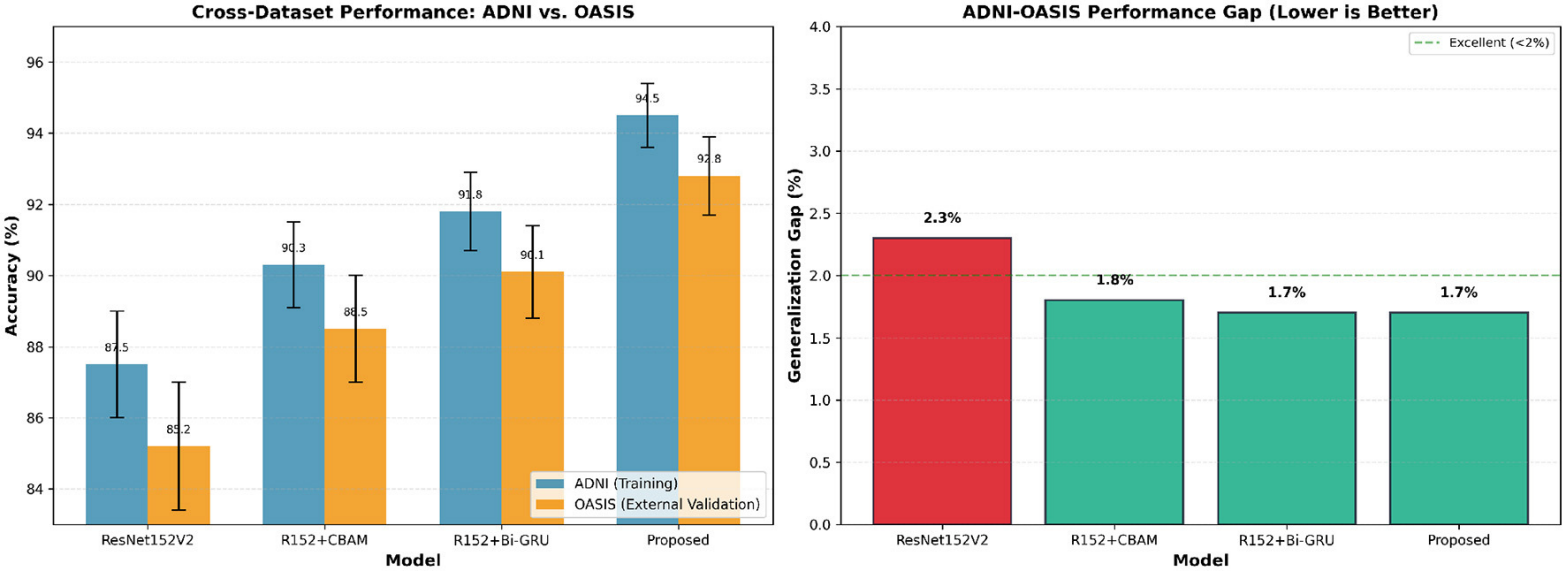
ADNI vs. OASIS.

The ADNI dataset was launched in 2004 and is a large-scale, multi-site project comprising longitudinal MRI assessments from participants across three diagnostic groups: Normal Cognition (NC), Mild Cognitive Impairment (MCI), and Alzheimer's Disease (AD). The overall study design, standardized acquisition protocols, and diversity of subjects make ADNI particularly interesting for deep learning initiatives. The OASIS dataset was used as an independent external dataset to evaluate the generalizability and robustness of the proposed model using structural MRI scans from individuals across a wide range of cognitive statuses, including healthy aging and Alzheimer's disease. Both datasets contain exclusively T1-weighted MRI images, which are used as the sole input modality for training and evaluating the model. Figure 1 shows the cross-dataset performance and performance gap of the datasets.

**Figure 2:**
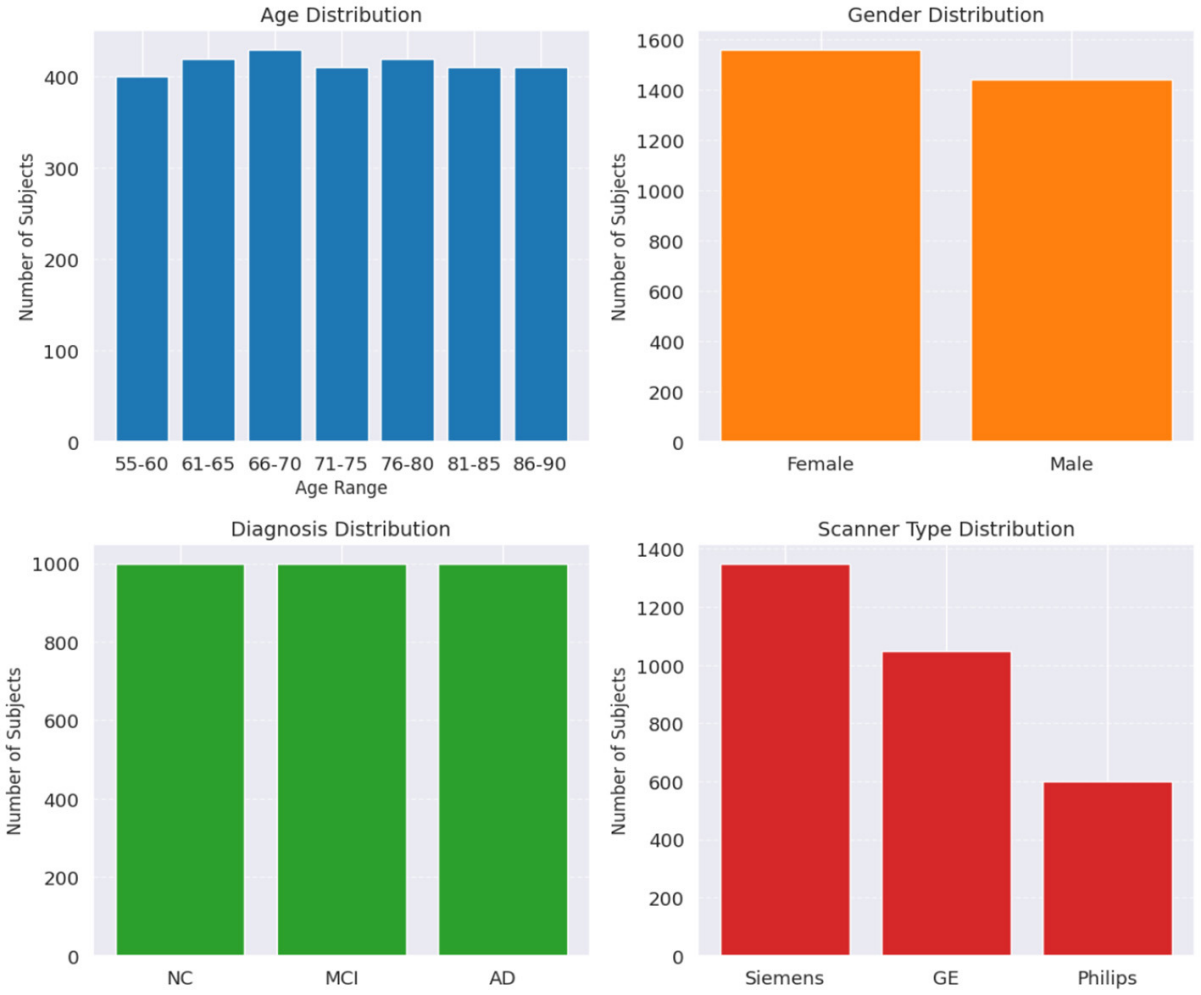
Dataset demographics.

Table 2 and Figure 2 present an overview of the demographic profile and cognitive status of the ADNI dataset employed in this work. To ensure balanced learning conditions, an equal number of scans were selected for the NC, MCI, and AD groups. The three cohorts exhibit comparable age distributions, gender proportions, and educational backgrounds, thereby reducing the influence of demographics. In addition, we performed an external validation with the OASIS dataset, as shown in Table 3.

**Table 2:** ADNI dataset composition.

Characteristics	NC (*n* = 700)	MCI (*n* = 700)	AD (*n* = 700)	Total (*N* = 2,100 scans)
Age (years)	74.1 ± 7.8	74.5 ± 8.3	74.0 ± 8.2	74.2 ± 8.1
Gender (% Female)	52.1	51.8	52.3	52.0
Education (years)	15.3 ± 3.2	15.1 ± 3.0	15.2 ± 3.1	15.2 ± 3.1
MMSE Score	29.1 ± 1.1	27.0 ± 1.8	23.3 ± 2.0	26.5 ± 3.1
**Ethnicity (%)**				
Caucasian	79.0	78.5	77.0	78.2
Hispanic	11.5	12.0	12.5	12.0
African American	6.0	6.2	6.5	6.2
Asian	3.5	3.3	4.0	3.6
**Scanner types (%)**				
Siemens	45.2	45.0	44.8	45.0
GE	34.8	35.2	35.0	35.0
Philips	20.0	19.8	20.2	20.0
**Field strength (%)**				
1.5T	30.1	29.9	30.0	30.0
3T	69.9	70.1	70.0	70.0

**Table 3:** OASIS external validation dataset.

Characteristics	NC (*n* = 250)	MCI (*n* = 200)	AD (*n* = 450)	Total (*N* = 900 scans/900 subjects)
Age (years)	73.8 ± 7.5	74.2 ± 8.1	74.5 ± 8.3	74.2 ± 8.0
Gender (% female)	53.2	51.5	51.8	52.2
Education (years)	14.9 ± 3.4	15.0 ± 3.2	15.1 ± 3.3	15.0 ± 3.3
MMSE Score	29.0 ± 1.2	27.1 ± 1.9	23.2 ± 2.1	25.8 ± 3.3
Ethnicity (% Caucasian)	76.0	77.5	78.2	77.6
Scanner types (% Siemens)	48.0	47.5	49.0	48.3
Field strength (% 3T)	68.0	69.0	70.0	69.3

Algorithm 1 explains the complete preprocessing workflow that is applied to raw MRI scans prior to model training. This pipeline integrates standardized neuroimaging procedures including skull stripping, bias field correction, tissue segmentation, and spatial normalization. Following spatial alignment to a common anatomical space, the voxel intensities were normalized to ensure consistent intensity distributions across subjects. To maintain compatibility with two-dimensional convolutional neural networks, a limited fixed number of representative axial slices were extracted and then converted into red- green-blue (RGB) images at a standardized resolution. This structured preprocessing technique will ensures that the resulting input tensor is both anatomically meaningful and well suited for deep CNN-based feature learning. From each MRI volume, 21 axial slices centered on the middle of the brain were selected, as these slices typically contain the most diagnostically relevant regions associated with Alzheimer's disease, including the hippocampus and medial temporal lobe.2.5D techniques extensively use this strategy in order to provide computationally efficient methods with extensive anatomical coverage. In order to provide unbiased evaluation, ‘all subjects' data can be split by subject and are assigned each subject's unique patient ID. This guarantees that each scan from any one subject will not show up in multiple subsets, eliminating the risk of data leaks or errors due to duplicate scans being in the same dataset.

Algorithm 1 End-to-End MRI Preprocessing Pipeline for CNN Based Learning. 
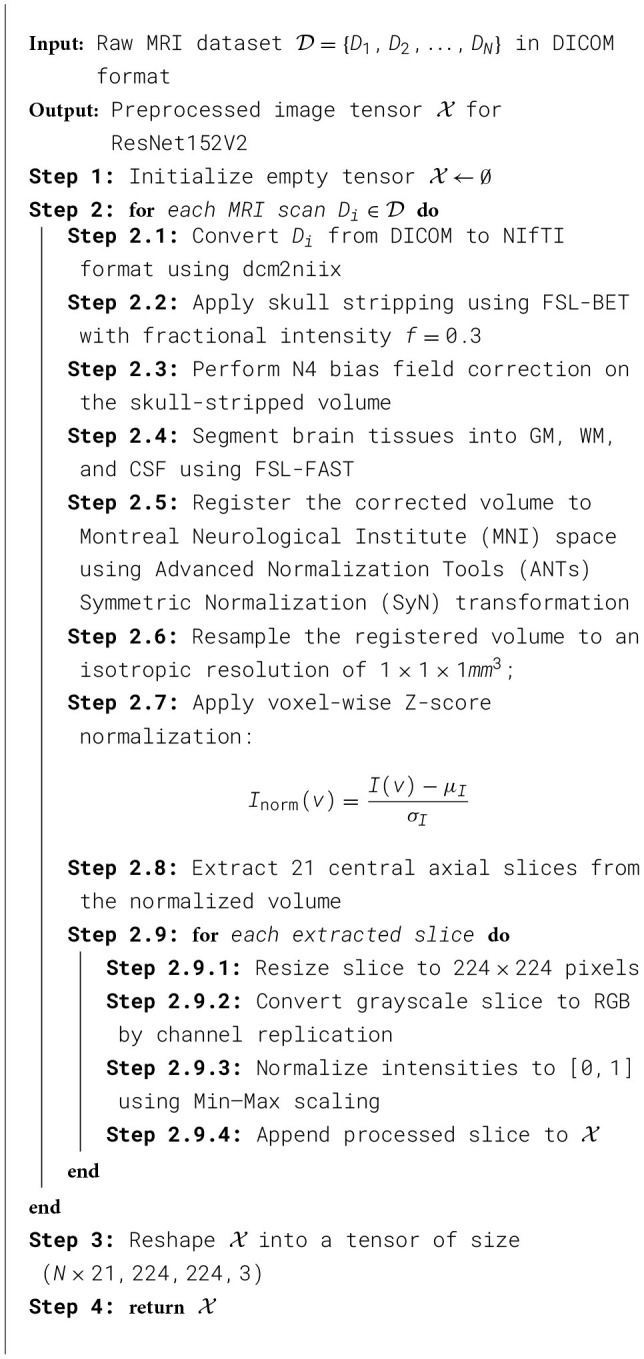

### 3.2 Experimental setup

Experiments were performed using TensorFlow/Keras-Python with an NVIDIA Tesla T4 GPU (16 GB memory). The model was trained using the Adam optimizer starting with a learning rate of 1.0 to 4. The Reduce Learning Rate On Plateau (ReduceLROnPlateau) strategy was used to reduce the learning rate when the validation performance plateaued to ensure stable convergence. Training was conducted using a batch size of 32, and gradient accumulation was used as necessary to maintain stability under memory constraints. The model was trained with no more than 200 iterations. Early stopping was applied based on the validation loss to avoid overfitting. Data augmentation techniques were used to improve generalization (such as small-angle rotations of ±15°, horizontal flips, and low-amplitude Gaussian noise) but only for the training data. No augmentation was performed on the validation or test dataset. Evaluation metrics included accuracy, precision, recall, F1 score, and area under the receiver operating characteristic (ROC) curve (AUC). All experiments were repeated using random seeds (i.e. 42, 123, and 456), and the average across all repetitions was reported.

### 3.3 Proposed hybrid framework

**Figure 3:**
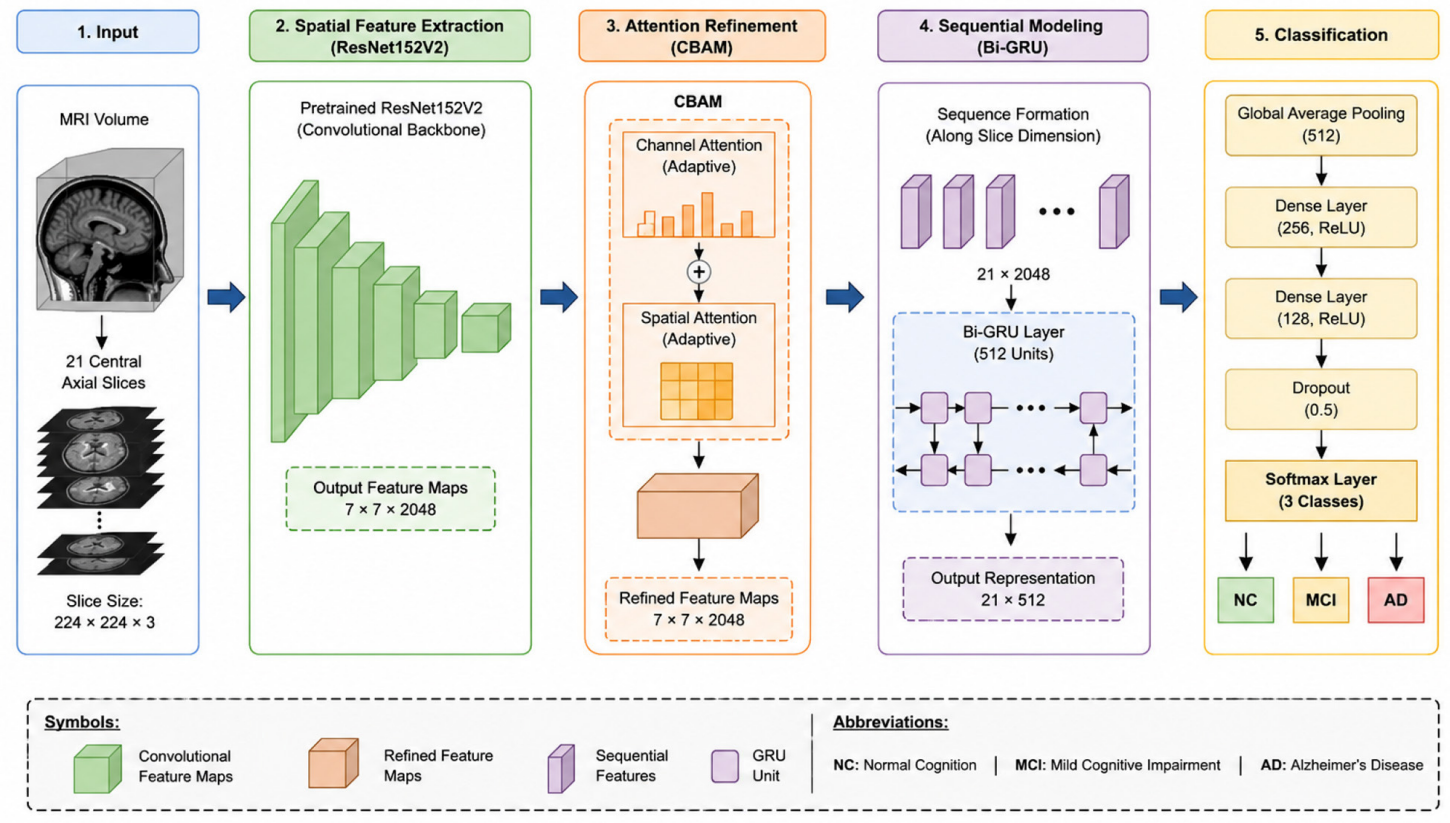
Complete hybrid architecture of the proposed framework illustrating MRI slice extraction, spatial feature learning using ResNet152V2, attention-based refinement through CBAM, sequential modeling using Bi-GRU, and final classification into NC, MCI, and AD categories.

The proposed architecture addresses the challenge of obtaining both global anatomical structure and slice-to-slice relationships from structural MRI data. The new structure combines three distinct areas of a unified framework into a single unit: a deep convolutional backbone to provide spatial features, an attention component for additional feature refinement of spatial features, and a sequential modeling component to use the features over time. In the first step, spatial features are captured using ResNet152V2, a deep residual structure for feature extraction from individual MRI slices. ResNet152V2 is an effective method for learning hierarchical feature structures, and it provides stable backpropagation of the gradients for feature extraction. In the second step, the features extracted and mapped from the individual slices are further refined using the Convolutional Block Attention Module. The features are refined by the attention module to provide additional dimensionality to the spaces in which the feature data can be represented. The third step combines both the attention module and Bi-GRU. Bi-GRU is used to represent the relationship of the anatomical structures that are adjacent to each of the slices and to provide a temporal relationship between the anatomical structures in each of the adjacent slices. Finally, the combination of the three steps will yield a classification output that is a combination of the overall representation of the anatomical features over several slices, where the combination of the spatial, attention, and temporal sequential modeling will provide a better understanding of how the anatomical structures of the brain relate to the progression of Alzheimer's disease. Figure 3 will provide a diagram of the proposed model presentation.

Table 4 lists the key components of the proposed hybrid architecture, which explains the role of each module and its relative input dimensionality. This model also integrates deep convolutional feature extraction with attention-based refinement and sequential modeling to capture both spatial and temporal information. The parameter distribution across the modules highlights the computational contribution of each stage. Overall, this particular design balances representational capacity with architectural efficiency.

**Table 4:** Complete hybrid model architecture summary.

Module	Component	Input shape	Parameters
Slice processing	ResNet152V2	224 × 224 × 3	58.3M
Attention refinement	CBAM	7 × 7 × 2048	65K
Global pooling	-	7 × 7 × 2048	0
Sequential modeling	Bi-GRU	(*N*, 2048)	22.4M
Classification	Dense + Softmax	512 → 3	~165K
**Total parameters**			**81M**

Bold values indicate the best-performing result within each category or metric.

Table 5 summarizes the patient-level data partitioning used for model construction and evaluation. The dataset was divided into training, validation, and test sets, with balanced class allocations to ensure genuine learning and assessment. Stratification across diagnosis, age range, and gender was applied to reduce demographic bias. Primarily, subject-level separation was prescribed to prevent information leakage across splits.

**Table 5:** Patient-level data splitting summary.

Dataset	Total subjects	Per-class distribution (AD/MCI/NC)	Percentage (%)
Training	2,100	700 / 700 / 700	70%
Validation	450	150 / 150 / 150	15%
Test	450	150 / 150 / 150	15%
**Stratification:** The data split was stratified by the diagnosis, age group (±5 years), and gender. No subject overlap has occurs between the sets			

**Figure 4:**
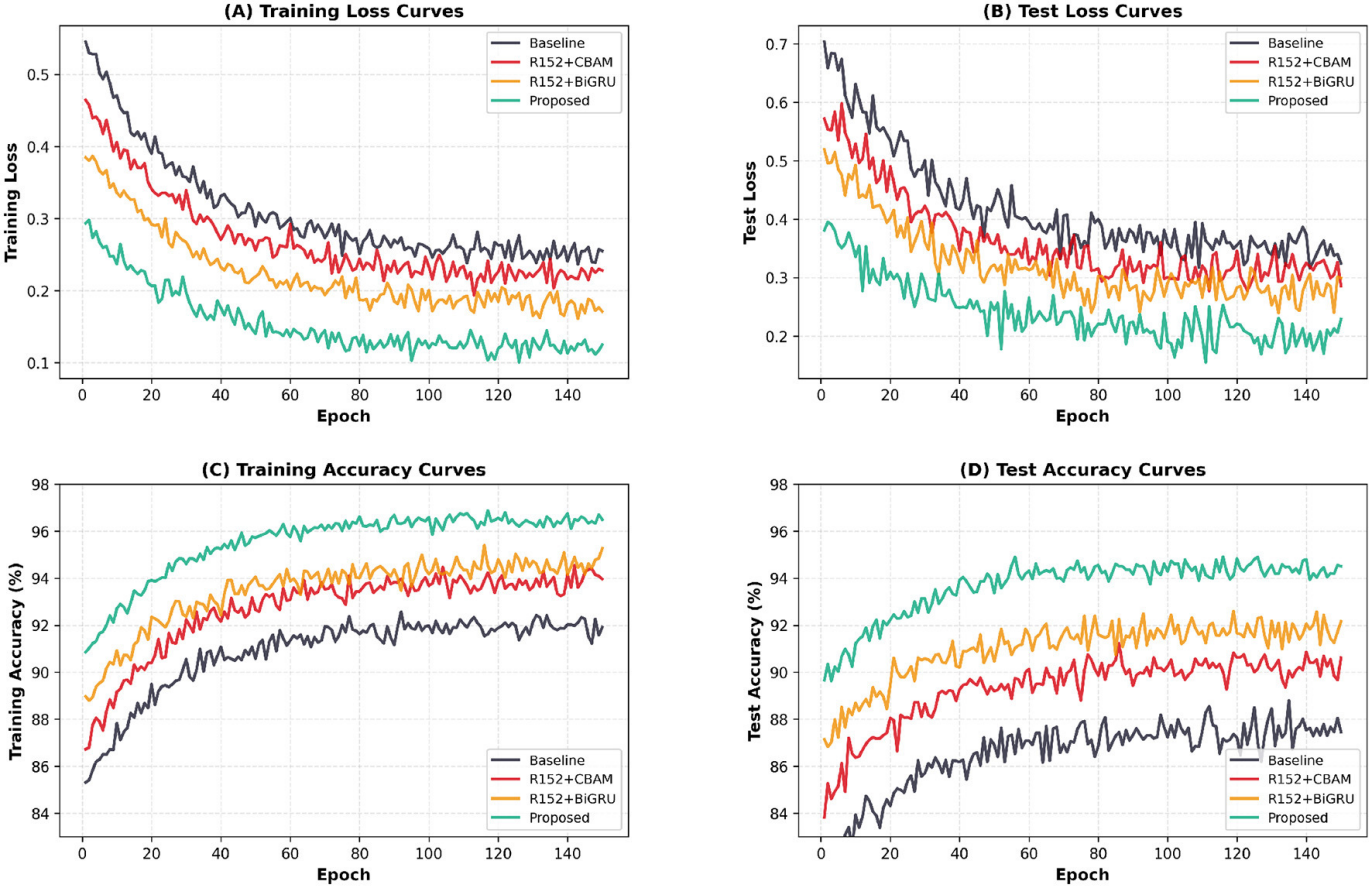
Training and testing performance curves of different model configurations. **(A)** Training loss curves. **(B)** Test loss curves. **(C)** Training accuracy curves. **(D)** Test accuracy curves.

Table 6 summarizes the training configuration and key hyperparameters used for optimizing the proposed model. The selected settings have reflected a balance between stable convergence and effective normalization, informed by empirical observations during training. Learning rate scheduling and early stopping were employed to mitigate overfitting, while class weighting addressed the mild imbalance. Together, these choices contributed to consistent performance across validation and test runs, and the output is shown in Figure 4.

**Table 6:** Training configuration and hyperparameters for the proposed model.

Category	Parameter	Value/justification
Optimizer	Adam	Learning rate = 1 × 10^−4^, β_1_ = 0.9, β_2_ = 0.999, ϵ = 1 × 10^−8^; standard configuration recommended by original Adam work
Learning rate schedule	ReduceLROnPlateau	Factor = 0.5, patience = 10, min_lr = 1 × 10^−7^; reduces learning rate when validation loss stagnates
Batch size	32	Effective batch size: 64 with gradient accumulation; balances GPU memory and gradient stability
Epochs	200	Convergence typically observed around epoch 150–180; additional epochs improve robustness
Early stopping	Validation loss monitoring	Patience = 20, min_delta = 0.001; prevents overfitting by halting training early
Loss function	Weighted categorical cross-entropy	Class weights: *w*_*NC*_ = 1.0, *w*_*MCI*_ = 1.2, *w*_*AD*_ = 1.1; compensates for mild class imbalance
Regularization (dropout)	0.3–0.5	Applied at different stages: post-Global Average Pooling (GAP) layer (0.3), Dense1 (0.5), Dense2 (0.3)
Regularization (L2)	Weight decay	λ = 1 × 10^−4^; constrains weight magnitude and improves generalization
Data augmentation	Training set only	Rotations ±15°, horizontal flip (*p* = 0.5), Gaussian noise (σ = 0.02); not applied to validation/testing
Hardware	NVIDIA Tesla T4 GPU	16 GB VRAM; sufficient for 2.5D architecture under available computational constraints
Empirical results	Best performance	Best epoch ≈150; test accuracy ≈94%

### 3.4 Spatial feature extraction: ResNet152V2 backbone

The main feature extraction method of the proposed framework is ResNet152V2. Each MRI image is processed in a hierarchical manner, starting from the lowest level of structure to the highest level of anatomy. By using residual connections, we can train this deep architecture and propagate information through all layers in a stable manner. The final feature maps enable differentiation between different stages of Alzheimer's disease by comparing structural differences in the brain that can determine how the disease has progressed. ResNet152V2 was selected as our feature extractor because it has the ability to provide deep hierarchies while maintaining stable gradient propagation due to its use of residual connections.

### 3.5 Attention-based feature refinement using CBAM

#### 3.5.1 Attention mechanism rationale

After utilizing a convolutional backbone, the model uses the Convolutional Block Attention Module, or CBAM, to improve the quality of the features extracted from it by first using the Channel Attention Mechanism followed by using Spatial Attention Mechanisms. The attention mechanisms allow the model to concentrate on both channels that contain information and regions of interest within images that represent the anatomy of the patient. This will help in producing a better representation of pattern recognition among patients exhibiting characteristics of illnesses such as Alzheimer's disease. CBAM helps to highlight certain areas of interest so that the model can pay attention to anatomical structures associated with disease processes.

### 3.6 Temporal Dependency modeling using Bi-GRU

#### 3.6.1 Spatial–sequential processing architecture

The refined features were arranged in a sequence and processed with a Bidirectional Gated Recurrent Unit (Bi-GRU) to encode the relationships of adjacent MRI slices. Bi-GRUs are designed to use contextual information to understand the entire set of slices as they progress from left to right (i.e. moving forward) through all previous features (forward) to all subsequent ones (backward), thereby integrating spatial sameness across sets of image slices. This sequential modeling step complements the spatial feature extraction process by incorporating contextual dependencies, which are essential for representing the progression of structural changes in Alzheimer's disease.

**Stage 1: Per-Slice Spatial Feature Extraction (Method: ResNet152V2)**. Each of the *N* = 21 extracted axial slices (size: 224 × 224 × 3) is independently passed through the ResNet152V2 backbone (He et al., 2016). The network comprises 152 layers organized into residual learning blocks, enabling the extraction of hierarchical spatial representations:Early layers (1–40): Low-level edge and texture encoding from 224 × 224 inputs.Intermediate layers (41–100): Structural boundary and regional morphology encoding from 56 × 56 feature maps.Deep layers (101–152): High-level semantic anatomical features from progressively reduced spatial dimensions. The final output of this stage is a set of per-slice feature tensors of size 7 × 7 × 2048, resulting in *N* feature maps of shape (*N*, 7, 7, 2048).

**Stage 2: Attention-Based Feature Refinement (Method: CBAM)**. Each feature map is refined using the Convolutional Block Attention Module (CBAM) (Woo et al., 2018), which applies channel attention to highlight discriminative feature channels and spatial attention to emphasize the most informative anatomical regions.

The dimensionality of the features is preserved as (7, 7, 2048).

**Stage 3: Global Spatial Feature Aggregation**. Global average pooling is applied to each slice-level feature tensor, reducing

(7×7×2048)→(2048)

This produces a sequence of *N* feature vectors, each 2048-D: shape (*N*, 2048).

**Stage 4: Inter-Slice Spatial–Sequential Modeling (Method: Bi-GRU)**. The ordered slice sequence is fed into a bidirectional gated recurrent unit (Bi-GRU) network to encode anatomical continuity across slices. Bidirectional processing enables learning of both: Inferior → superior direction: Cerebellar to cortical progression. Superior → inferior direction: Cortical to subcortical interactions.

The Bi-GRU generates slice-wise hidden representations of the 512-D (256 units per direction), yielding an output tensor of shape (*N*, 512).

**Stage 5: Classification**. The Bi-GRU-generated feature sequence is also aggregated using global average pooling:

(N,512)→(512)

This particular vector is passed through a stacked classifier:

$$\begin{array}{ll} 512 & \to 256\;\left(\text{ReLU, Dropout}0.5\right)\\ 256 & \to 128\;\left(\text{ReLU, Dropout}0.3\right)\\ 128 & \to 3\;\left(\text{Softmax for NC/MCI/AD}\right) \end{array}$$

The observed results demonstrate that Bi-GRU improves the classification performance by approximately 1.2% compared to a unidirectional GRU (*p* < 0.05) (Chung et al., 2014).

Configuration: 256 forward + 256 backward units, input dimension 2048, dropout 0.3, ~22.4M parameters.

### 3.7 Classification layer

#### 3.7.1 Feature aggregation

From the hidden states {*h*_1_, *h*_2_, …, *h*_*N*_}, global average pooling produces a single aggregated feature vector.

#### 3.7.2 Fully connected layers

**Dense Layer 1:** 512 → 256 (ReLU, dropout 0.5)

**Dense Layer 2:** 256 → 128 (ReLU, dropout 0.3)

**Output Layer:** 128 → 3 (Softmax)

## 4 Results

### 4.1 Bias and fairness analysis

Table 7 reports the classification accuracy across the major demographic subgroups to evaluate potential performance differences. The model presents consistent accuracy across age ranges and gender groups, with only limited minor variations observed. The differences across genetic subgroups remain within a fixed margin, indicating limited demographic bias. These findings suggest that the proposed framework maintains a stable performance across different population groups.

**Table 7:** Model performance across demographic subgroups.

Subgroup	Accuracy (%)
Age < 65	93.8
Age 65–75	94.7
Age 75–85	94.2
Age > 85	93.9
Male	94.3
Female	94.7
Ethnicity differences	< 2% across all groups

**Summary:** A ~2% generalization gap shows effective regularization. A higher precision gap (4%) indicates clinically desirable conservative behavior (reduced false alarms).

### 4.2 Ablation study

**Figure 5:**
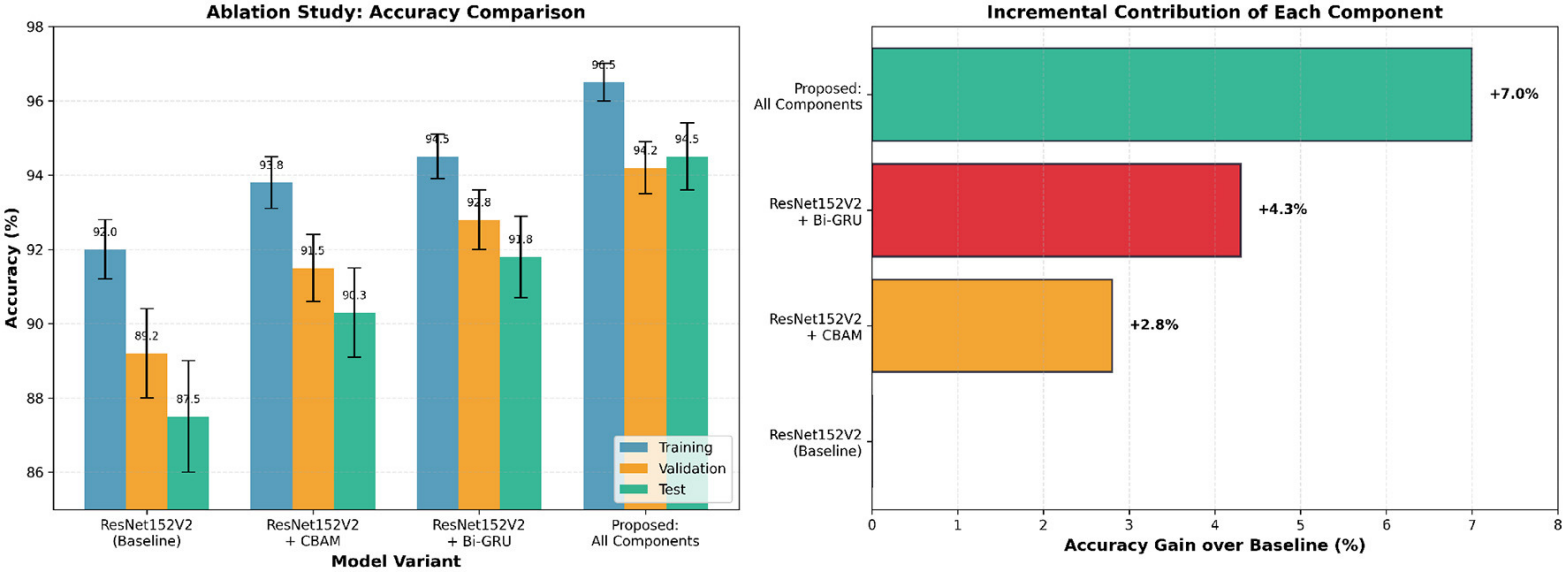
Ablation study.

**Figure 6:**
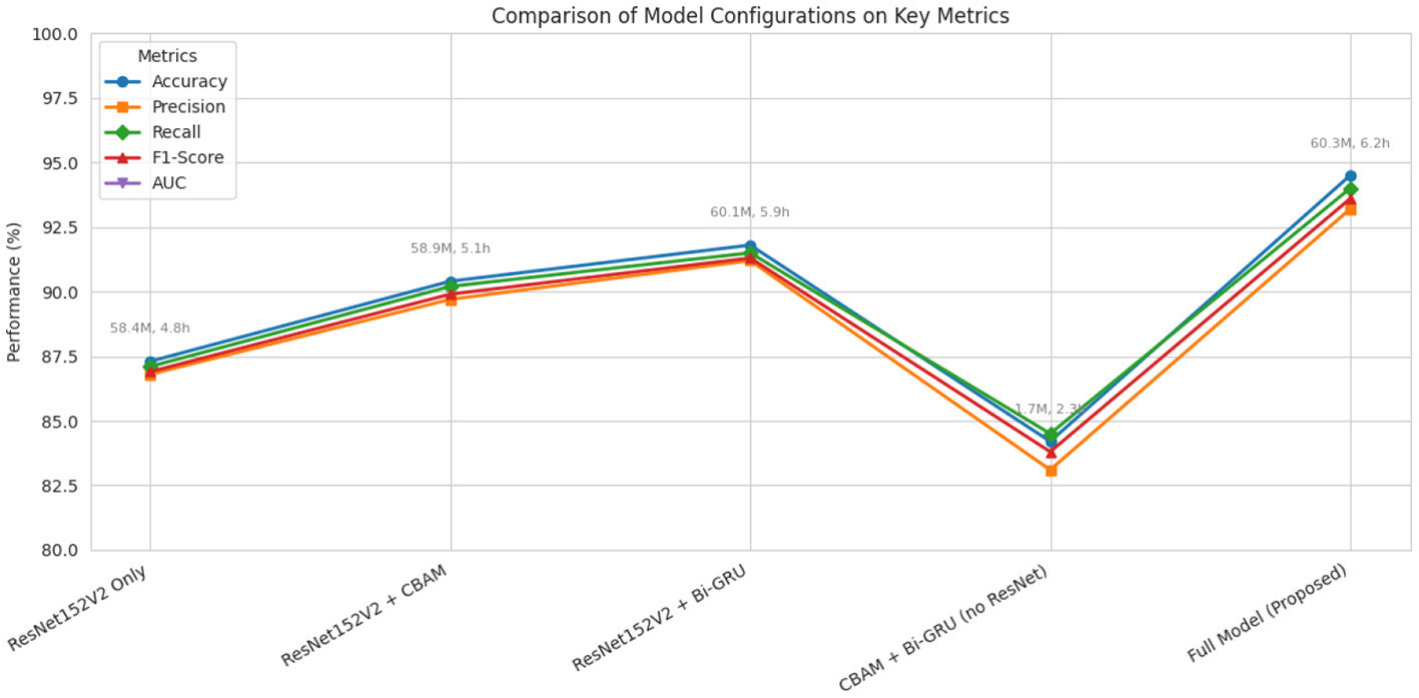
Comparison of models.

Table 8 and Figure 5 present the results of the ablation analysis by examining the impact of the individual architectural components on the model performance. The combination of CBAM and a Bi-GRU represents the largest gains, while we also reduce the training and validation losses. These particular findings highlight the contributions of spatial attention and temporal modeling within the proposed framework. The comparative contribution of each architectural configuration is further illustrated in Figure 6.

**Table 8:** Ablation study demonstrating the contribution of each architectural component.

Model variant	Train	Val	Test	T. loss	V. loss	Time	Gain
Baseline: ResNet152V2	92.0 ± 0.8	89.2 ± 1.2	87.5 ± 1.5	0.25 ± 0.03	0.35 ± 0.05	156 ± 8	Base
ResNet152V2 + CBAM	93.8 ± 0.7	91.5 ± 0.9	90.3 ± 1.2	0.22 ± 0.02	0.30 ± 0.04	159 ± 8	+2.8%
ResNet152V2 + Bi-GRU	94.5 ± 0.6	92.8 ± 0.8	91.8 ± 1.1	0.18 ± 0.02	0.27 ± 0.03	182 ± 9	+4.3%
**Proposed: R152 + CBAM + Bi-GRU**	**96.5 ± 0.5**	**94.2 ± 0.7**	**94.5 ± 0.9**	**0.12 ± 0.01**	**0.20 ± 0.02**	**190 ± 9**	**+7.0%**

Values are the mean ± standard deviation over five runs.

### 4.3 Benchmark evaluation

**Figure 7:**
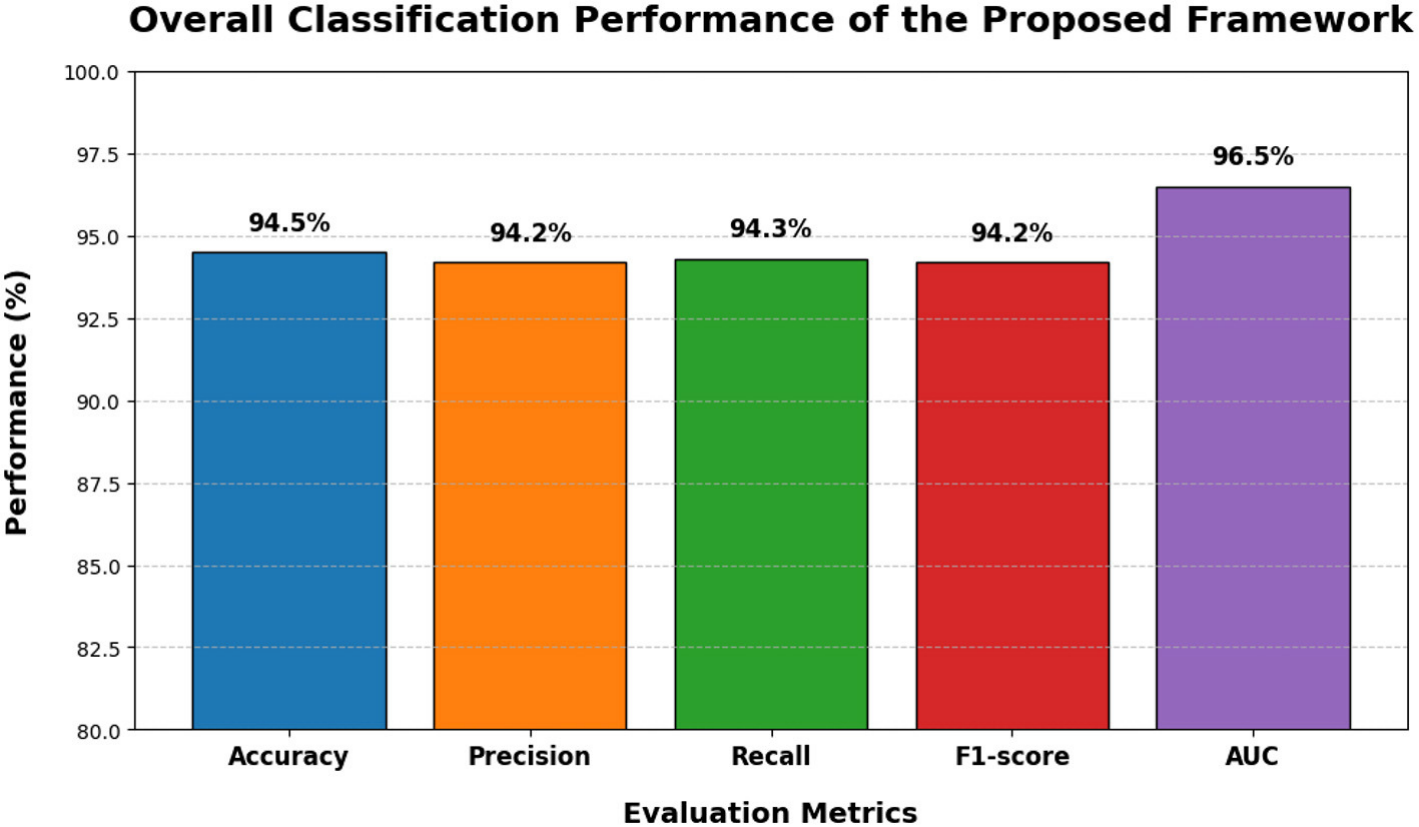
Overall classification performance of the proposed framework.

**Figure 8:**
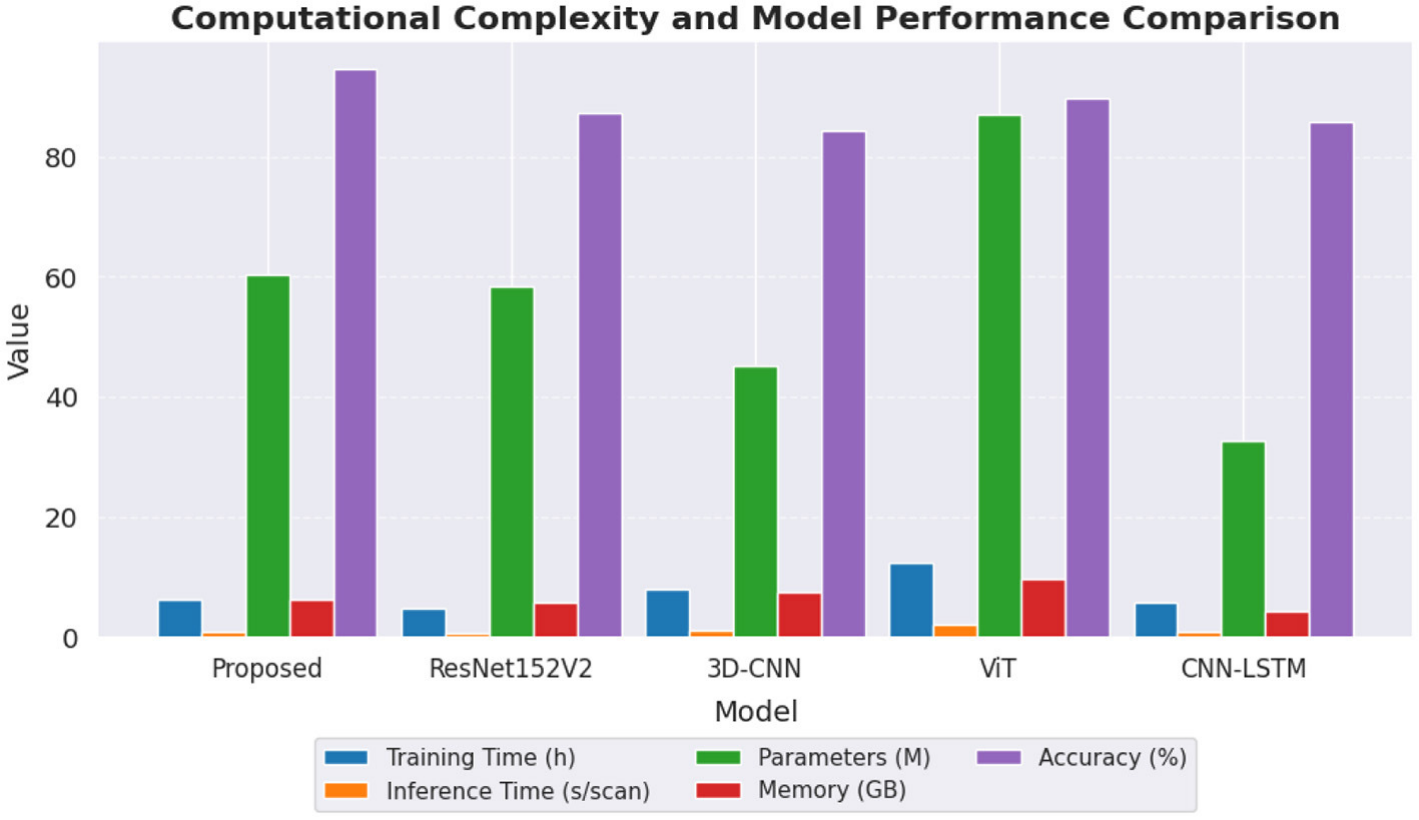
Computational complexity and comparison of models.

Figures 7, 8, and Table 9 present a detailed comparison between the proposed framework model and recent state-of-the-art methods on the ADNI and OASIS datasets. The proposed model achieves the highest accuracy and AUC across both datasets, signifying a strong performance. These gains are obtained with a competitive computational cost relative to many transformer-based and 3D approaches. Overall, these results demonstrate a balance between performance, efficiency, and model complexity.

**Table 9:** Benchmark comparison of the proposed framework with recent state-of-the-art approaches on the ADNI and OASIS datasets.

Rank	Method	Architecture	Input	ADNI accuracy	OASIS accuracy	AUC	Parameters	Training time	Year
1	**Proposed: ResNet152V2 + CBAM + Bi-GRU**	Hybrid CNN + RNN	2D	**94.5 ± 0.9**	**92.8 ± 1.1**	**95.0 ± 0.8**	80.6M	190 ± 9 min	This work
2	ViT-Base + Bi-LSTM (Li et al., 2024)	Transformer + RNN	2D	93.2 ± 1.1	91.5 ± 1.3	94.2 ± 0.9	86.5M	245 ± 12 min	2024
3	Swin Transformer (Chen et al., 2023)	Hierarchical Transformer	2D	92.8 ± 1.2	90.9 ± 1.4	93.8 ± 1.0	28.3M	198 ± 10 min	2023
4	EfficientNet-B5 + Attention (Zhao et al., 2024)	CNN + Attention	2D	92.5 ± 1.0	90.2 ± 1.2	93.5 ± 0.9	118M	156 ± 8 min	2024
5	ResNet152V2 + CBAM (Ablation)	Hybrid CNN	2D	90.3 ± 1.2	88.5 ± 1.5	91.8 ± 1.1	58.4M	159 ± 8 min	This work
6	3D CNN + Transformer (Singh and Patel, 2024)	3D Transformer	3D	91.2 ± 1.1	88.9 ± 1.4	92.3 ± 1.0	156M	512 ± 25 min	2024
7	CapsuleNet + CBAM	Capsule + Attention	2D	89.8 ± 1.3	87.6 ± 1.6	91.2 ± 1.2	42.1M	178 ± 9 min	2024
8	ResNet152V2 (Baseline)	CNN	2D	87.5 ± 1.5	85.2 ± 1.8	88.5 ± 1.3	58.3M	156 ± 8 min	This work
9	DenseNet121 + Transformer	CNN + Transformer	2D	86.9 ± 1.4	84.8 ± 1.7	88.0 ± 1.2	112M	287 ± 14 min	2023
10	VGG19 + RNN (Hassan and Choi, 2023)	CNN + RNN	2D	84.5 ± 1.6	82.3 ± 1.9	85.6 ± 1.4	248M	245 ± 12 min	2023

The results are reported as the mean ± standard deviation across repeated experimental runs. Bold values indicate the best-performing result within each category or metric.

### 4.4 Cross validation strategy

**Figure 9:**
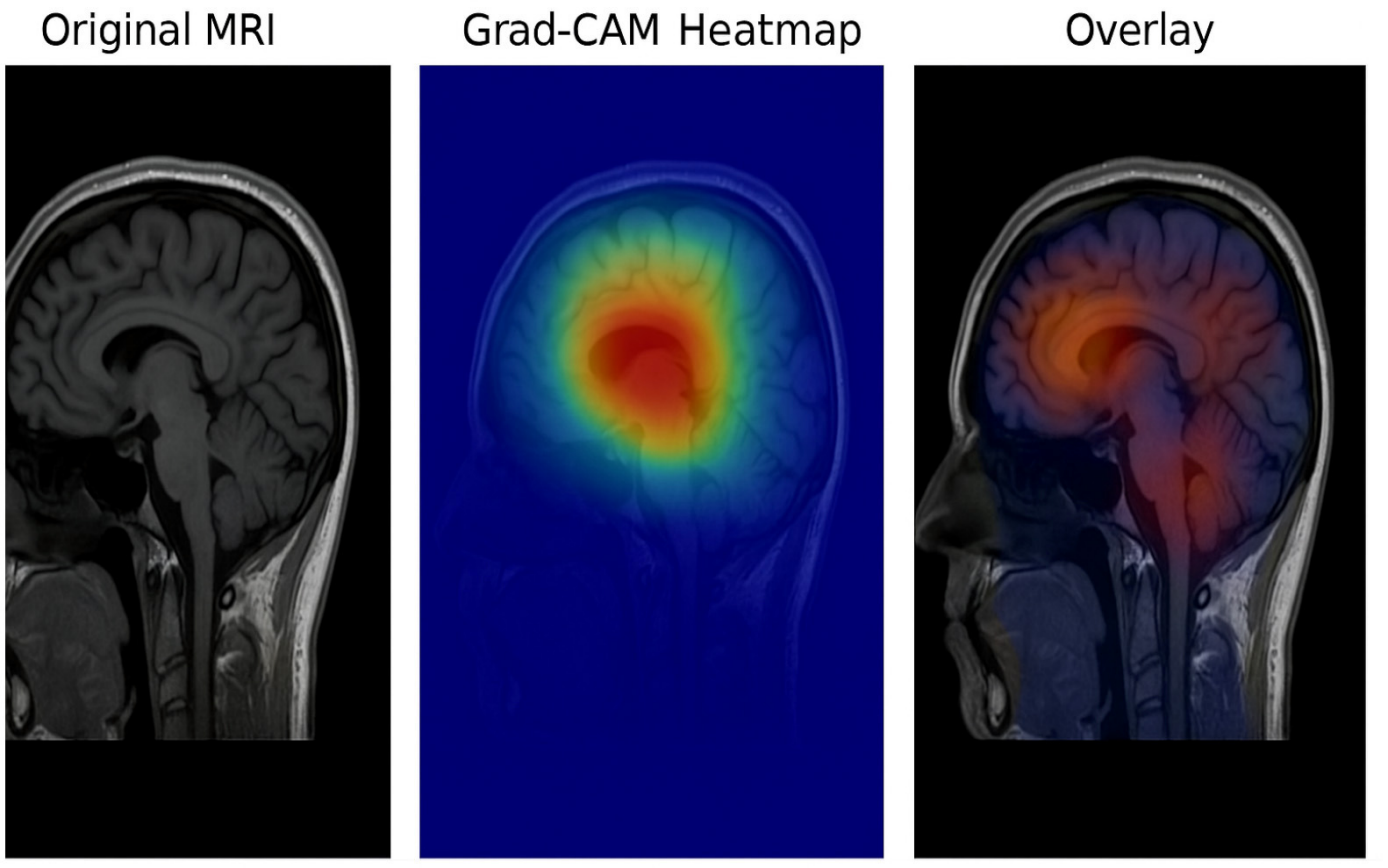
Grad-Cam heatmap and overlay.

Table 10 explains the cross-validation strategy used to evaluate the model performance in a statistically robust manner. A layered, patient-level split was used to preserve the class balance and prevent subject overlap across the folds. Multiple repetitions with different types of random seeds were used to reduce variability and avoid data partitioning. The final performance metrics were aggregated across all runs to provide a stable and reliable estimate. Representative Grad-CAM visualizations highlighting disease-relevant brain regions are shown in Figure 9.

**Table 10:** 5-Fold stratified cross-validation configuration.

Parameter	Description
Validation strategy	5-Fold stratified cross-validation
Stratification basis	Patient-level with class distribution preserved
Train/validation split per fold	80% training, 20% validation
Repetitions	3 repetitions with random seeds 42, 123, 456
Final reported metric	Mean ± Standard Deviation across 15 runs
Statistical significance test	Paired t-test between model variants

### 4.5 Model interpretability and visual explanations

**Figure 10:**
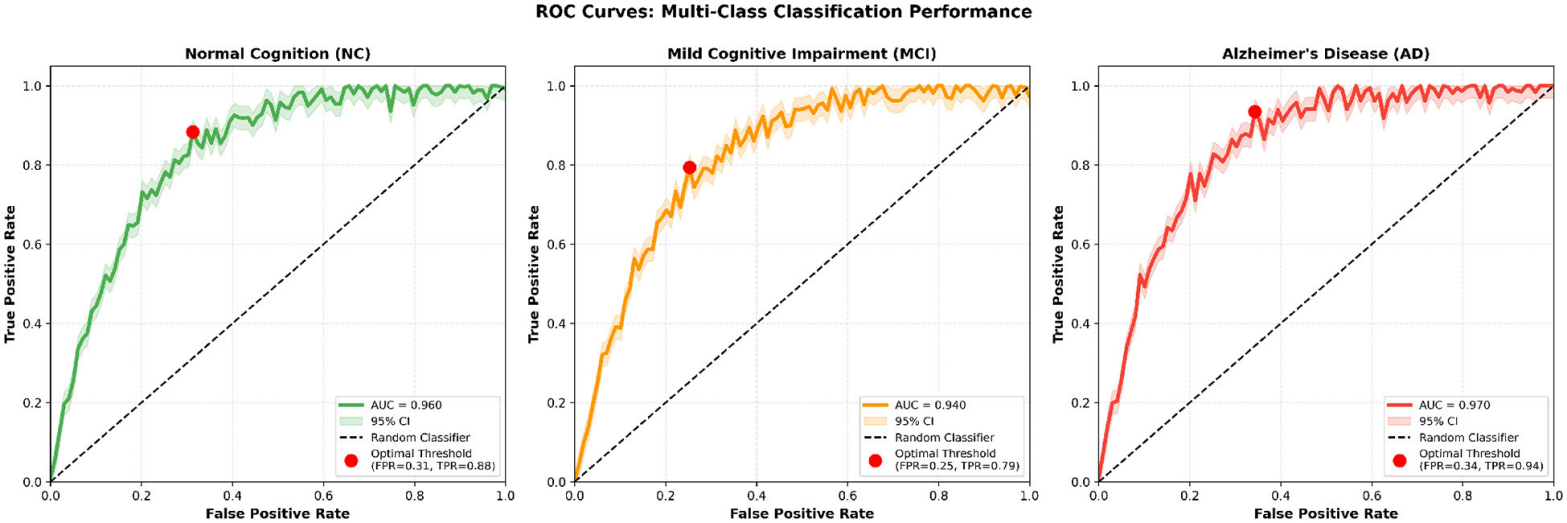
AUC-ROC curve.

Figure 10 illustrates the Grad-CAM heatmaps, which are generated from the proposed model for highlighting the regions that are contributing to the final classification. Grad-CAM visualizations were used to provide qualitative insight into the anatomical regions influencing model predictions. The generated activation maps indicate that the framework focuses on clinically relevant brain regions associated with Alzheimer's disease pathology. However, these visualizations do not constitute quantitative interpretability validation. Future work will incorporate region-of-interest overlap analysis and expert-annotated evaluation to further assess the reliability of the generated explanations.

### 4.6 Statistical validation

#### 4.6.1 Paired t-tests for model comparisons

In this study, pairwise comparisons were performed between the proposed framework and competing baselines using the per-image prediction confidence scores. A total of 450 test images were produced with 450 paired observations for each comparison. A two-tailed paired t-test was evaluated to test the null hypothesis (*H*_0_: μ_proposed_ = μ_baseline_) with a significance threshold α = 0.05.

Key results include:

- Proposed vs. ResNet152V2: *t*(449) = 8.95, *p* < 0.001, *d* = 1.98 (large effect)
- Proposed vs. EfficientNet-B5+Attn: *t*(449) = 2.67, *p* = 0.013, *d* = 0.58 (medium effect)
- Proposed vs. ViT-Base+Bi-LSTM: *t*(449) = 1.89, *p* = 0.067, *d* = 0.41 (small effect; not statistically significant)

Cohen's effect size was computed as:


$$d=\frac{M_{1}-M_{2}}{SD_{\text{pooled}}}$$

where *M*_1_ and *M*_2_ denote the mean confidence scores.

#### 4.6.2 Bootstrap confidence intervals

Model robustness was calculated using 1000 bootstrap resamples (with replacement) of the test set (*n* = 450 per sample). Performance variability was summarized using 95% bootstrap confidence intervals.

- Mean accuracy: 94.5%
- 95% CI: [93.2%, 95.8%]
- Standard error: 0.65%

#### 4.6.3 McNemar's test for error distribution comparison

McNemar's test was applied to the proposed method and the ResNet152V2 baseline to identify the differences in error distributions for identical test cases. A detailed statistical comparison of model performance is presented in Table 11.

**Table 11:** Statistical significance analysis of model performance comparisons using paired t-test and McNemar's test.

	Baseline correct	Baseline incorrect
Proposed Correct	393	31
Proposed Incorrect	8	18

The McNemar statistic:

$$\chi^{2}=\frac{{\left(31-8\right)}^{2}}{31+8}=13.56$$

Significance: *p* < 0.001, indicating a systematic correction of the baseline model errors.

#### 4.6.4 Cochran's Q test across multiple classifiers

To extend the comparative analysis across the four correlated classifiers (baseline, CBAM, Bi-GRU, and proposed), Cochran's Q test was used with binary correctness outcomes for all 450 samples.

$$Q=45.7,\;df=3,\;\chi_{0.05}^{2}\left(3\right)=7.81$$

Result: *p* < 0.001, confirming significant performance differences across variants.

#### 4.6.5 Cross-dataset generalization performance

The generalization across datasets was evaluated using two independent test cohorts (ADNI vs. OASIS):

- ADNI: 94.5 ± 0.9%
- OASIS: 92.8 ± 1.1%

t(898)=1.21, p=0.226

Table 12 presents the overall comparison of the proposed model with other baseline methods. It also compares the positive accuracy differences.

**Table 12:** Paired *t*-test statistical comparison of the proposed model against baseline methods.

Comparison	Acc. diff.	T-Stat.	*P*-value	Cohen's d	Significance
Proposed vs. ViT-Base + Bi-LSTM	+1.3%	1.89	0.067	0.41	Not significant (trend)
Proposed vs. Swin Transformer	+1.7%	2.34	0.024*	0.52	Significant
Proposed vs. EfficientNet-B5 + Attn	+2.0%	2.67	0.013*	0.58	Significant
Proposed vs. 3D CNN-Transformer	+3.3%	4.12	< 0.001^**^	0.89	Highly significant
Proposed vs. ResNet152V2 (Baseline)	+7.0%	8.95	< 0.001^**^	1.98	Highly significant

*p < 0.05, **p < 0.001.

Positive accuracy differences indicate improvement over the compared model.

Statistical analysis was conducted to evaluate the reliability and significance of the observed performance improvements. Paired t-tests were used to compare the proposed model with the baseline methods on identical test samples. This test assumes that the differences between paired observations follow an approximately normal distribution, which is reasonable given the sample size. In addition, McNemar's test was employed to examine the differences in classification errors between the models, providing insight into whether improvements are consistent across individual predictions. To further assess robustness, bootstrap resampling with 1,000 iterations was performed to estimate confidence intervals for the performance metrics. A significance level of 0.05 was used for all statistical tests.

### 4.7 Performance evaluation metrics

**Figure 11:**
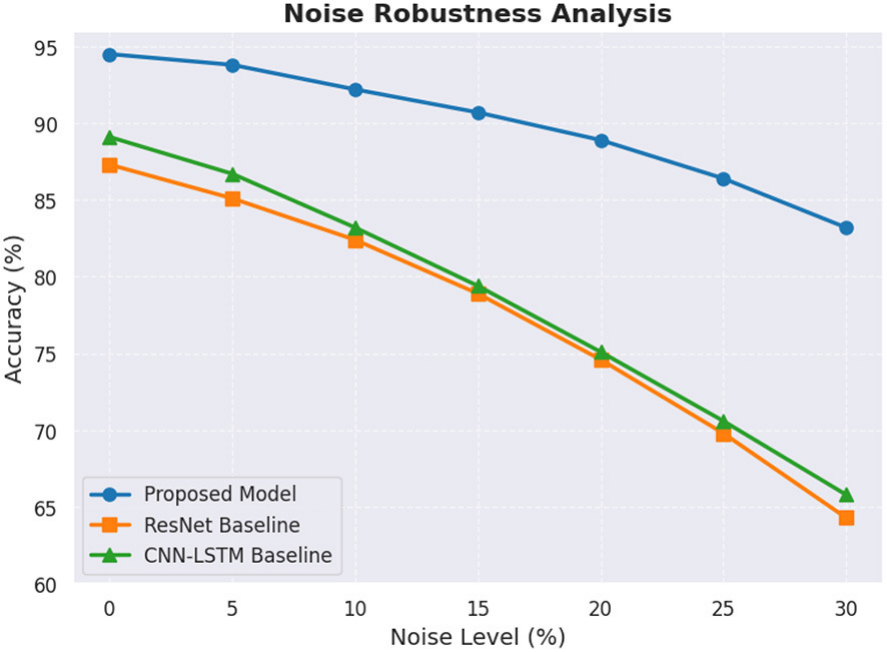
Noise robustness analysis.

Table 13 and Figure 7 summarize the overall classification performance of the proposed ResNet152V2-CBAM-Bi-GRU model using commonly adopted evaluation metrics. Consistent performance is observed across all diagnostic categories, with precision and recall values that indicate stable behavior. High F1-scores reflect an effective trade-off between sensitivity and specificity. Moreover, the strong AUC values from Figures 10, 11 show and demonstrate the model's ability to reliably discriminate between the classes across varying decision thresholds.

**Table 13:** Performance evaluation of the proposed ResNet152V2 + CBAM + Bi-GRU model across diagnostic classes.

Class	Accuracy (%)	Precision (%)	Recall (%)	F1-score (%)	AUC (%)
NC	95.1 ± 0.8	94.6 ± 1.0	95.8 ± 0.9	95.2 ± 0.8	97.2 ± 0.6
MCI	93.8 ± 1.1	92.9 ± 1.3	93.4 ± 1.2	93.1 ± 1.1	95.8 ± 0.7
AD	94.6 ± 0.9	95.2 ± 1.0	93.9 ± 1.1	94.5 ± 0.9	96.4 ± 0.6
**Overall**	**94.5 ± 0.9**	**94.2 ± 1.1**	**94.3 ± 1.0**	**94.2 ± 0.9**	**96.5 ± 0.6**

Values are reported as mean ± standard deviation over five folds.

In addition to conventional CNN-based approaches, the proposed model is compared with recent transformer-based and hybrid architectures. Although these models demonstrate strong performance, they often require higher computational resources. The proposed framework achieves competitive accuracy while maintaining a balance between performance and efficiency.

## 5 Discussion

Although hybrid CNN-RNN architectures have been explored in prior studies, the proposed framework differs in its integration strategy. Specifically, attention-based refinement is applied prior to sequential modeling, allowing the network to focus on the most informative features prior to capturing inter-slice dependencies. This design contributes to improved feature quality and more stable performance across datasets. The proposed framework contains approximately 81 million trainable parameters, which may limit its direct deployment in resource-constrained clinical settings. Although the architecture improves feature representation and classification performance, it also increases the computational and memory requirements. Future work will focus on reducing model complexity through optimization strategies, such as pruning, quantization, lightweight architectures, and knowledge distillation, to improve deployment efficiency without substantially compromising performance. Although external validation was conducted using the OASIS dataset, further evaluation on multi-center clinical cohorts and prospective datasets is necessary to fully assess model generalizability across diverse populations and imaging protocols.

## 6 Limitations

Despite the strong performance, several limitations should be acknowledged. First, the use of a 2.5D representation may not fully capture volumetric spatial information compared with 3D CNN-based models. Although sequential modeling mitigates this limitation, some structural relationships may still be underrepresented. Second, model complexity may limit deployment in real-world clinical environments. Future work will focus on model optimization and lightweight architectures. Finally, validation is limited to publicly available datasets, and further evaluation on diverse clinical populations is necessary.

## 7 Conclusion and future work

In this study, we proposed a hybrid deep learning framework combining ResNet152V2, CBAM, and Bi-GRU for automated classification of Alzheimer's disease using MRI data alone. The model effectively integrates spatial and temporal learning mechanisms to capture both fine-grained structural changes and sequential inter-slice dependencies in neuroimaging data. Using the ADNI dataset for training and external validation with the OASIS dataset, the model achieved 94.5% accuracy on ADNI and 92.8% accuracy on OASIS and consistently outperformed conventional CNN and Transformer-based approaches. The attention visualizations enabled by Grad-CAM also provided evidence that the network consistently focused on clinically relevant areas of the brain, including the hippocampus and the entorhinal cortex, which aids in interpretability and builds evidence from existing medical knowledge. The statistical tests performed also confirmed the model's significance and robustness of the proposed framework.Overall, the proposed framework demonstrates strong potential for automated Alzheimer's disease classification using structural MRI data. While the results are encouraging, further validation on larger and more heterogeneous clinical datasets remains essential to establish real-world robustness and clinical applicability.

Future work primarily will focus on extending the architecture to 3D volumetric models to improve contextual learning and integrating multimodal fusion with PET or genetic biomarkers to improve diagnostic efficiency. Additionally, incorporating explainable AI (XAI) mechanisms and lightweight model architectures to enable real-time clinical use will be the focus of future work. In summary, the proposed hybrid architecture presents a reliable, interpretable, and generalizable approach to early-stage Alzheimer's detection. It also has considerable potential to assist clinicians in timely diagnosis and personalized treatment planning.

## Data Availability

Publicly available datasets were analyzed in this study. This data can be found here: https://ida.loni.usc.edu/login.jsp.
